# Pericytes Contribute to Dysfunction in a Human 3D Model of Placental Microvasculature through VEGF‐Ang‐Tie2 Signaling

**DOI:** 10.1002/advs.201900878

**Published:** 2019-10-29

**Authors:** Kristina Haase, Mark R. Gillrie, Cynthia Hajal, Roger D. Kamm

**Affiliations:** ^1^ Massachusetts Institute of Technology Cambridge MA 02139 USA; ^2^ Department of Medicine University of Calgary Calgary AB T2N 1N4 Canada; ^3^ Present address: EMBL Barcelona Carrer del Dr. Aiguader, 88 Barcelona Spain 08003

**Keywords:** angiogenesis, pericytes, placenta, pre‐eclampsia, vascular dysfunction

## Abstract

Placental vasculopathies are associated with a number of pregnancy‐related diseases, including pre‐eclampsia (PE)—a leading cause of maternal–fetal morbidity and mortality worldwide. Placental presentations of PE are associated with endothelial dysfunction, reduced vessel perfusion, white blood cell infiltration, and altered production of angiogenic factors within the placenta (a candidate mechanism). Despite maintaining vascular quiescence in other tissues, how pericytes contribute to vascular growth and signaling in the placenta remains unknown. Here, pericytes are hypothesized to play a detrimental role in the pathogenesis of placental vascular growth. A perfusable triculture model is developed, consisting of human endothelial cells, fibroblasts, and pericytes, capable of recapitulating growth and remodeling in a system that mimics inflamed placental microvessels. Placental pericytes are shown to contribute to growth restriction of microvessels over time, an effect that is strongly regulated by vascular endothelial growth factor and Angiopoietin/Tie2 signaling. Furthermore, this model is capable of recapitulating essential processes including tumor necrosis factor alpha (TNFα)‐mediated vascular leakage and leukocyte infiltration, both important aspects associated with placental PE. This placental vascular model highlights that an imbalance in endothelial–pericyte crosstalk can play a critical role in the development of vascular pathology and associated diseases.

## Graphical abstract

A model of perfusable placental microvasculature recapitulates vessel remodeling in 3D, altered barrier function, and infiltration by polymorphonuclear neutrophils—key observations associated with placental vasculopathies, such as those often present in pre‐eclampsia. Here, endothelial–pericyte interactions significantly impact vessel remodeling, with pericytes causing growth restriction—a process regulated by vascular endothelial growth factor and the Ang/Tie2 signaling axis.

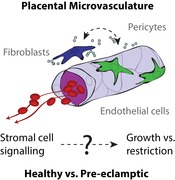

## 1 Introduction

**Figure 1:**
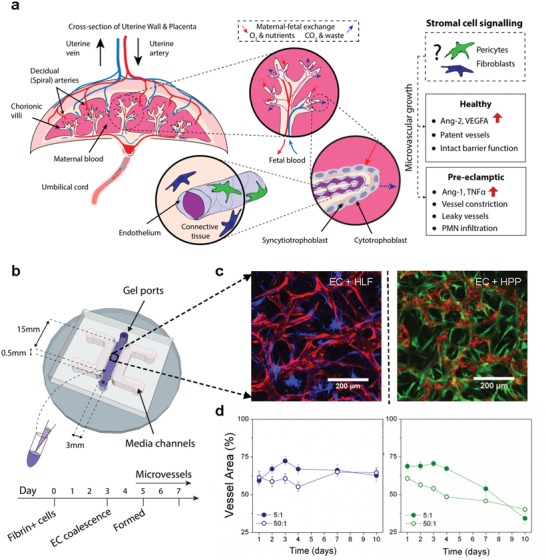
Endothelial and stromal cell coculture for generation of 3D microvessels in a macroscale platform. a) Diagram of the placenta and vasculature in the fetal terminal villi. The role of stromal cells, aside from trophoblasts, in vessel development and regulation remains largely unknown. b) Schematic diagram of the macroscale PDMS device used to generate microvessels encapsulated in single‐channel of fibrin gel. Cells are cultured together and form connected microvessels by day 5 in the fibrin gel. They are generally maintained over 1–2 weeks. c,d) Stromal cells are shown at day 4 of culture with HUVEC (red), for c) HLF cocultures (blue —HLFs), and HPP cocultures (green—HPPs). d) Mean vessel area coverage is shown for 2 ratios of EC:stromal cells for HLF cocultures (left—blue) and HPP cocultures (right—green). Shown is mean ± s.e.m.

Pre‐eclampsia (PE) is the most prominent disease leading to maternal–fetal morbidity and mortality worldwide, the only cure for which is delivery.^1^ While some pre‐existent maternal conditions cause PE with normal placentation, a severe form termed “placental” PE is associated with abnormal placentation^2^ and four‐ to sevenfold higher incidence of vasculopathy than normal pregnancies.^3^ The placental‐phenotype of this disease is largely attributed to vascular dysfunction of the placenta, the organ that serves as the master regulator of maternal–fetal exchange of oxygen, nutrients, and waste (**Figure** **1**a). Reduced vascular perfusion, increased oxidative stress, increased neutrophil recruitment, and an altered angiogenic response have all been observed in clinical presentations of this disease; however, the exact cause remains unknown.^4^ Insufficient trophoblast invasion of maternal spiral arteries is one proposed cause of PE that has dominated the field; however, an imbalance of angiogenic signals has also been proposed as the driving mechanism of this disease. PE is concomitant with significantly altered plasma markers—decreased angiogenic factors (vascular endothelial growth factor (VEGF), placental growth factor (PLGF), and Angiopoietin‐2 (Ang‐2)) and increased soluble antiangiogenic factors (soluble fms‐like tyrosine kinase‐1 (sFlt‐1), and soluble endoglin (sEng)).^4^ Angiogenesis in vitro has also been shown to be drastically altered by culture with plasma from patients with PE.^5^ Given that endothelial cells and pericytes are well‐known to regulate angiogenesis during the later stages of gestation, it is possible that dysfunctional pericyte–endothelial signaling could contribute to the development of placental vasculopathies, such as those in PE.

Pericytes are typically associated with vascular stabilization (reviewed in^6^); however, their role in vascular pathobiology,^7^ and specifically their role in the placenta remains largely unknown. Placental pericytes have been suggested to act as regulators of fetal angiogenesis,^8^ with expression of associated markers (α‐smooth muscle actin (α‐SMA) and platelet‐derived growth factor receptor‐β (PDGFR‐β)) having been shown to increase from the chorionic plate to terminal villi during early placentation (7–8 weeks).^9^ Vessel coverage by pericytes has also been shown to significantly increase from the first to the last trimester (≈40–60%), the gestational age at which PE symptoms typically appear.^10^ Considering that the surface area of villi increases throughout gestation as demands on maternal–fetal nutrient exchange increase, angiogenic regulation in this region is vital. Recent investigations have demonstrated reciprocal Angiopoietin/Tie‐2 signaling between endothelial cells and pericytes,^11^ suggestive of a complex interplay that might arise between these heterotypic cells during vessel growth.

Considering its importance in fetal development, many models have been used in an effort to elucidate placental pathophysiology, including PE.^12^ The distinct differences between human and other animal models,^13^, ^14^ and the limited time within which explants can be employed (≈6 h), have made adoption of tissue‐engineering strategies for the development of in vitro placenta models more attractive.^15^, ^16^ Recent models have focused on the role of trophoblasts in regulating transport across the maternal–fetal barrier using a cell‐layering or transwell approach.^17^, ^18^ However, the possibility to develop more physiological model systems certainly exists, including bio‐engineering tissues with functional 20–50 µm diameter microvessels surrounded by tissue‐relevant stromal cells including pericytes.^19^, ^20^, ^21^ Tissue engineering approaches provide a critical tool to dissect cell‐specific influences on blood vessel formation, growth, and function. Despite these recent advances, little has been done to explore the potential for pericyte regulation of microvessel growth or restriction in a human 3D model of placental microvasculature.

Here, we develop a 3D in vitro model of placental terminal villi microvessels, to specifically examine placental pericyte‐mediated angiogenic signaling imbalances and microvascular dysfunction. Moreover, we use this model to examine placental vascular inflammation, endothelial barrier dysfunction, and leukocyte–endothelial interactions. Our model recapitulates key characteristics of an inflamed microvascular environment, similar to PE. Importantly, placental pericytes are shown to contribute to growth restriction and endothelial barrier dysfunction in this microenvironment. Specifically, we find that fibroblast pro‐angiogenic signaling is negatively regulated by antiangiogenic Tie‐2/Ang‐2 placental pericyte signaling, the latter of which can be overcome by the addition of exogenous fibroblasts growth factor (FGF) and VEGF. Our results provide a new model for the study of placental vasculopathies (such as those seen in some cases of PE) and suggest that pericytes could be contributors to the endothelial dysfunction concomitant with this disease, where leaky vessels, increased neutrophils, and irregular angiogenic signaling dominate. Finally, this human 3D model of placental microvessels highlights that agonists of VEGF‐ and FGF‐signaling may be beneficial in preventing dysfunctional placental angiogenesis.

## 2 Results and Discussion

### 2.1 Placental Pericytes Inhibit Microvascular Growth

To date, no models of perfusable placental microvasculature exist—yet these are necessary to explore the role of stromal cells in placental vascular dysfunction. The human placenta contains various stromal cells (Figure 1a), including trophoblasts, pericytes, and fibroblasts, the two latter of which are known to regulate vascular growth and function in other organs (reviewed in^22^). Fetal‐derived trophoblasts have been the focus of previous 2D models of PE;^17^, ^18^ however, little attention has been placed on the possible role of pericytes or fibroblasts in PE‐associated vascular dysfunction.^23^, ^24^ Consistent with their importance in vessel function, our group and others have shown that fibroblasts and pericytes are necessary for the maintenance and long‐term growth of in vitro 3D microvessels.^20^, ^21^, ^25^ To develop a placental microvasculature model of the terminal villi, we designed an in vitro macroscale fluidic device which allows for the coculture of different combinations of primary human stromal cells alongside endothelial cells (ECs) within a central 3D fibrin hydrogel (Figure 1b). Using similar microscale devices, we have shown that microvessel formation occurs in a process akin to vasculogenesis (neo‐vessel formation) within 1 week and allows for morphological and functional studies of human microvasculature.^21^, ^26^

After design and fabrication of the devices (see^27^ for details), we set out to establish successful 3D in vitro microvessel generation in the presence or absence of human placental pericytes (HPPs). To generate and monitor microvessels, we utilized red fluorescent protein‐labeled primary human umbilical vein endothelial cells (HUVEC) of fetal origin to visualize vascular morphology. As a source of placental stromal cells, we used green fluorescent protein (GFP)‐expressing primary HPPs, and previously employed human lung fibroblasts (HLFs), as a source of placental fibroblasts was not readily available at the onset of this study. Immunofluorescence staining of stromal cells showed that both HLFs and HPPs express αSMA and NG2 (Figure S1a, Supporting Information), which are commonly used as perivascular smooth muscle and pericyte markers, respectively.^28^ As a baseline, we confirmed that HUVEC do not form sustainable networks alone (Figure S1b, Supporting Information) but rather require a coculture, at a seeding ratio of 5:1, with HLFs.^21^ As expected, coculture of HUVEC with HPPs at a seeding ratio of 5:1 showed that HPPs aligned with microvessels similar to HLFs (Figure 1c) and HPPs were found to reside within the basement membrane of ECs (Figure S1c, Supporting Information) consistent with previous reports in other tissues.^28^ Furthermore, HUVEC‐HPP cocultures generate connected structures consistent with vascular networks; however, total vascular area per device was significantly reduced by the presence of placental pericytes as compared to HUVEC‐HLF cocultures (46 ± 4% vs 67 ± 4% area on day 7, *P* = 0.002, for *n* = 7 and *n* = 6 devices, respectively).

Considering that stromal cell density has been linked to EC turnover and varies over the course of placentation,^10^ we employed different endothelial‐stromal cell ratios to investigate differences in vasculogenesis between HPP and HLF cocultures (Figure 1d). Regardless of initial seeding density, HLFs resulted in stable vessels defined as consistent vessel area coverage (% ECs projected in 2D) after 10 d of culture. On the other hand, a reduction in HPP seeding density from 1.2 to 0.12 × 10^6^ cells mL^−1^ continued to result in restricted and largely disconnected vessels over time. Regardless of initial seeding density, we did find that placental pericytes proliferated within the 3D microvascular environment throughout culture (Figure S2a,b, Supporting Information), and could be recruited by ECs (Figure S2d–f, Supporting Information).

**Figure 2:**
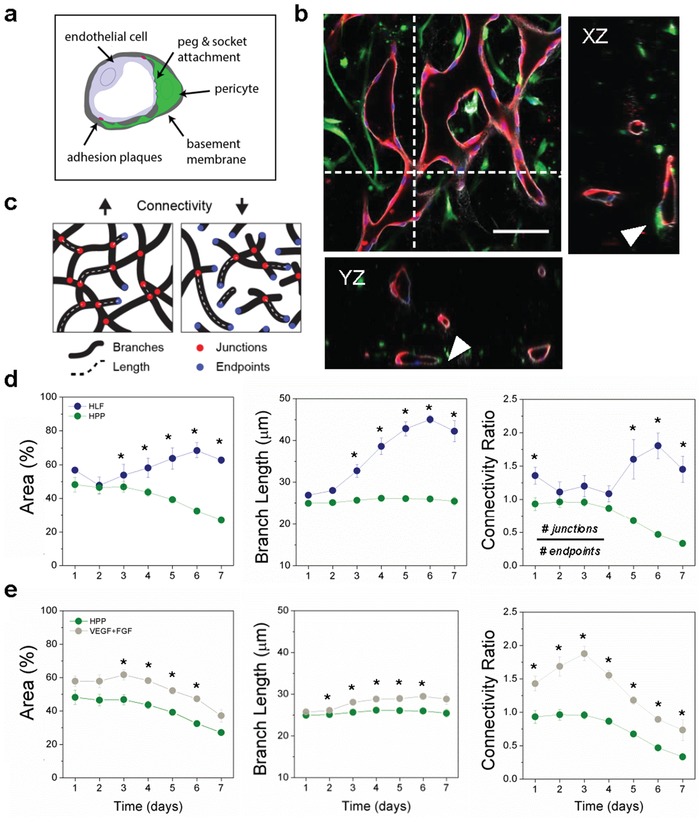
Placental pericytes reduce microvessel growth and connectivity. a) Schematic diagram showing pericyte location in relation to microvessels in vivo. b) Confocal image of HPP‐cocultured with HUVEC fixed at day 5. Shown is a single XY plane and orthogonal projections demonstrating lumen (red) wrapped by HPPs (green), as indicated by white arrows. Nuclei were labeled with Dapi (blue). Scale bar is 200 µm. c) Schematic showing the various geometric measurements using binary projection images. d) Comparison of mean vessel area (EC coverage), branch length, and microvessel connectivity between HLF and HPP cocultures. Significant differences between parameters appear early on. e) Parameters are compared for HPP cocultures with (green) and without (gray) added VEGF+FGF. Shown is mean ± s.e.m. *P > 0.05 with *t*‐test.

Placental pericytes wrap around vascular endothelial cells as expected (**Figure** **2**a,b); notably they appear to constrict microvessels over longer culture durations. Thus, a number of morphologic parameters were used to compare vascular growth between the two cocultures (Figure 2c). Representative data from multiple experiments show drastic differences in vascular area, branch geometry, and overall network connectivity between HPP and HLF cocultures, as observed daily by confocal microscopy. Notably, HPP coculture results in many disconnected microvessels, as indicated by a reduced connectivity ratio (Figure 2d). Similar decreases in vessel density and diameter have been shown with HUVEC‐pericyte cultures in 3D angiogenesis assays.^25^, ^29^

We postulated that differences between stromal cell interactions and vascular growth could be due to cell specific differences in cytokine expression. Media collected and pooled from devices were tested by cytokine array to examine differences between the two coculture conditions (Figure S3a, Supporting Information). Of note, inflammatory cytokines and chemokines (GROα, TIMP‐1, IL‐6, IL‐8, MCP‐1) were strongly expressed, with IL‐6 decreasing over time (from day 3 to 5) for both cocultures (in the presence of complete media). Expression of these inflammatory cytokines occurs during normal pregnancy, but their over‐expression is strongly associated with pathologies, such as PE.^30^ However, no major differences between HPP or HLF cocultures in classic PE‐associated angiogenic cytokines were found, including VEGFA or bFGF.

Considering the prominence of VEGFA and bFGF in early vascular development (in particular of mesenchymal villi) and their deficit in PE,^31^ both proangiogenic (vessel forming) cytokines were employed exogenously in an effort to promote microvessel growth and maintenance in HPP‐HUVEC cocultures. While these growth factors (GFs) improved vascular growth for HPPs in the initial days following seeding, their effect was insufficient for generating connected vasculature and resulted in a decreased connectivity ratio over time (Figure 2e). At the same concentration, these GFs had no apparent influence on HLF cocultures (Figure S3b, Supporting Information). These results suggest that placental pericytes can maintain fetal vascular structures during early vasculogenesis, but have a negative impact on longer‐term (>3 d) vascular morphology versus fibroblast cocultures. Importantly, even with the addition of exogenous VEGFA and bFGF we were unable to obtain perfusable microvessels of HPP cocultures (Figure S4a, Supporting Information), limiting our ability to explore features of PE such as altered endothelial permeability.

### 2.2 A Triculture Model of Pre‐Eclamptic‐Like Microvessels

**Figure 3:**
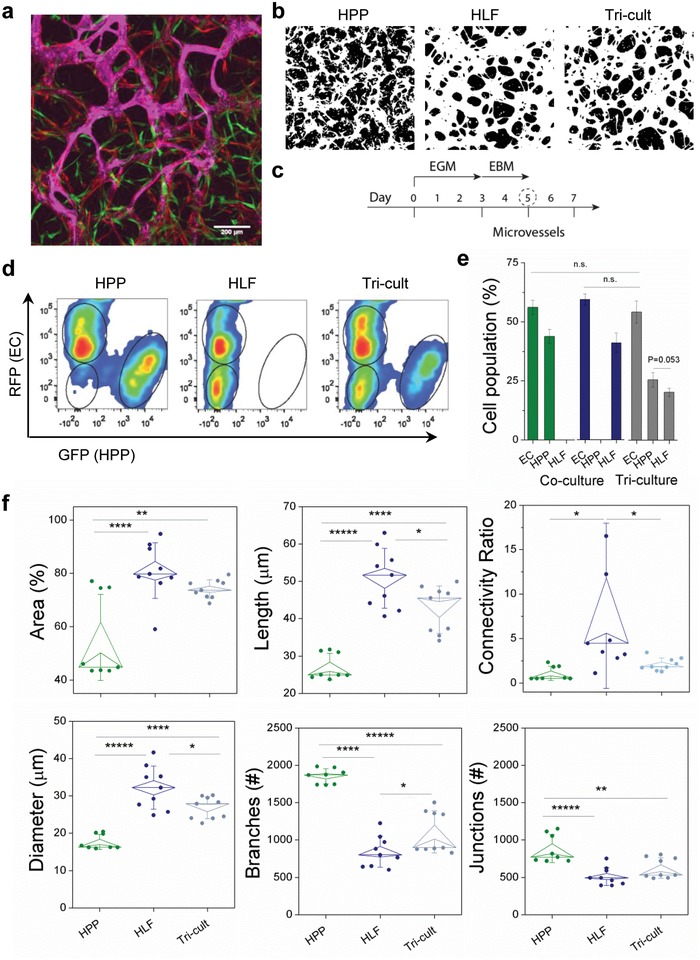
A triculture model for increased microvessel connectivity. a) A triculture microvascular system perfused with fluorescently labeled beads. HUVEC—red, pericytes—green, 10 µm beads—magenta. b) Binary images from maximum intensity projections for co‐ and tricultures, as shown at day 5. c) Schedule for media change from full growth endothelial growth medium (EGM) to reduced serum basal medium (EBM). d) Representative flow cytometry density plots for HPP, HLF, and tricultures. e) Mean population of ECs and stromal cells for the co‐ and tricultures at day 5, as measured by flow cytometry. Three separate devices for each culture condition were used for measurement and repeated in *n* = 3 separate experiments. f) Microvessel parameters are compared between co‐ and tricultures. Shown is mean ± s.e.m. Significance is indicated by *P < 0.05, **P < 0.01, ***P < 0.001, ****P < 0.0001, one‐way ANOVA and Tukey test.

Considering that fibroblast cocultures result in stable in vitro vessels but pericyte cocultures do not, we attempted a triculture model to generate perfusable microvessels so that we could interrogate the role of placental pericytes in microvascular function. Pericytes are known to change phenotype and have close phenotypic relations to mesenchymal stem cells.^32^ Therefore, we first characterized our placental pericytes by flow cytometry, demonstrating expression of both smooth muscle (αSMA and calponin) and pericyte‐specific markers (NG2 and PDGF‐Rβ) in 2D and 3D cultures (Figure S5, Supporting Information). By including HLFs in triculture, HPP containing microvessels were patent, as demonstrated by fluorescent beads perfused through 50–75% of the triculture vessels, demonstrating their interconnectivity and increased vessel coverage compared to cocultures (**Figure** **3**a,b). Given that our previous results suggested exogenous cytokines could positively influence vessel morphology (Figure 2e), tricultures and subsequent HPP‐ or HLF‐HUVEC cocultures were grown in complete media until day 3 (to allow for initial EC coalescence) and subsequently switched to basal media + 1%FBS (Figure 3c). This allowed us to mechanistically explore the direct effects of stromal cells in the absence of exogenous GFs. The seeding ratio of ECs to stromal cells was kept consistent for the triculture (5:1), but with 50% each of HPPs and HLFs (final ratio 5:0.5:0.5). Following 5 d in culture, changes in the percentage of ECs and stromal cell populations were assessed by flow cytometry (Figure 3d). For co‐ and tricultures, HPPs and HLFs proliferated similarly, and much more than ECs. Their growth resulted in a near 1:1 ratio of ECs to stromal cells by day 5 (10:8 on average, across co‐ and tricultures) (Figure 3e). This proliferative capacity is reminiscent of increased proliferation of stromal cells derived from PE placentas,^33^ and suggests that a balance between stromal cells types and their proliferative capacity may contribute to placental vascular growth and dysfunction, the mechanisms of which are not yet known, but are under current investigation.

To characterize vessels in triculture versus HPP‐ or HLF‐ cocultures, a detailed comparison of microvessel geometry in different culture conditions was performed using confocal microscopy. By day 5 postseeding, tricultures consistently improved microvessel growth, as demonstrated by increased EC% area coverage within each field of view, microvessel length, and connectivity ratio (ratio of vessel junctions to endpoints), compared to HPP cocultures (Figure 3f). Importantly, the presence of HPPs in the triculture resulted in significantly reduced vessel diameters in comparison to HLF cocultures, again demonstrating the role of pericytes in restricting vessel lumen diameter. Together these results establish that the triculture model including HPP, HLF, and HUVEC yields perfusable microvascular networks with narrowed vessels that are responsive to cytokine alterations. While we cannot recapitulate all aspects of placental microvasculature, the features of our model allows us to study key signaling pathways and vessel function in more detail to understand what events may be dysregulated in placental vasculopathies, such as those seen in PE.

### 2.3 Pericytes Mediate Vessel Growth through VEGF‐Tie2 Signaling

**Figure 4:**
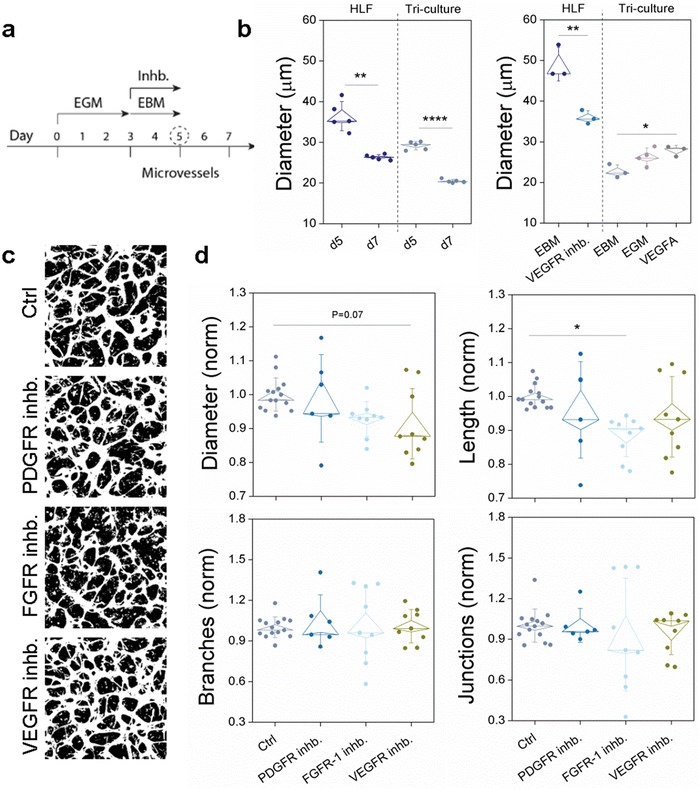
Placental pericytes contribute to microvessel dysfunction. a) Schedule of inhibitor and culture conditions. b) Mean vessel diameters shown for HLF cocultures and tricultures over time, and in full growth factor media (EGM) and basal media (EBM), with and without VEGFA stimulation or inhibition. *P < 0.05, **P < 01, ***P < 0.001, ****P < 0.0001 with paired *t*‐test. c) Representative binary images of microvessels are shown at day 5 for the various treatments indicated. d) Microvessel morphologic parameters are compared for the various treatments. In order to minimize experiment variability, normalization was performed by dividing by the mean control value for that particular experiment. Significance is indicated by *P < 0.05, one‐way ANOVA with Tukey test.

Signaling pathways, including PDGF, Notch, bFGF, VEGFA, and Angiopoietin/Tie2, that have been implicated in placental vascular growth (reviewed in^30^, ^34^), have also been suggested as either a cause or primary effect of placental PE through their dysregulation. Here, we employed a variety of inhibitors to establish whether these pathways regulate growth of our placental microvessels (**Figure** **4**). Tricultures were grown in basal media supplemented with inhibitors for 48 h (re‐established daily) prior to imaging. First, considering that patency of vessels is affected by GFs in our system (Figure S4a, Supporting Information) we characterized vessel remodeling in response to VEGFA and full GF containing media. Significant remodeling (as witnessed by changes in vessel diameter) occurred between 5 and 7 d for both HLF co‐ and tricultures in complete media (Figure 4b). Vessel diameter was also increased by the addition of exogenous VEGFA in HPP containing tricultures (Figure 4b). Although PDGFβ (stimulated by Ang‐1) is well‐known to direct pericytes toward ECs and is critical for normal vascular development in the placenta,^13^ chemical inhibition of PDGF receptors (PDGFR) with AG 1296 had no effect on either microvessel morphology (Figure 4c,d) or pericyte coverage (data not shown). PDGF signaling in our triculture model may be confounded due to early proximity of pericytes and ECs prior to drug administration negating the impact of PDGFβ/PDGFR signaling on pericyte recruitment to microvessels.

Given that our earlier results demonstrated that exogenous bFGF and VEGFA positively impact vascular growth in HPP‐HUVEC cocultures (Figure 2e), and that they are well‐known to promote angiogenesis,^34^ we moved to examine the role of their respective receptors on vascular morphology. In contrast to the lack of effect of PDGFR inhibition, FGFR and VEGFR chemical inhibitors (PD‐166 866 and SU5416, respectively) both affected microvessel diameter (although variability between experiments was high), with FGFR inhibition significantly reducing vessel length (Figure 4d). VEGF is known to be severely downregulated in PE^35^ which corresponds well with our results showing that exogenous VEGFA can improve vessel morphology in placental pericyte‐containing co‐ and tricultures (Figure 4b). Correspondingly, treatment with sFLT‐1 (commonly over‐expressed in PE and an inhibitor of VEGFA activity) led to significant vessel regression, even in the presence of VEGFA (Figure S6, Supporting Information). Whether or not pericytes affect VEGF receptor expression including sFLT‐1 levels in the placenta, which are highly upregulated in PE,^36^ remains to be seen, but this could be one cause of endothelial dysfunction.

**Figure 5:**
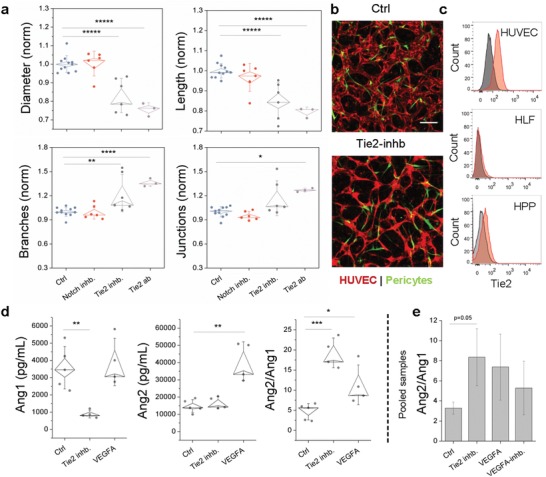
Ang‐Tie2‐Vegf signaling largely regulates microvessel morphology. a) Comparison of parameters between triculture microvessels treated with various inhibitors. Shown is mean ± s.e.m. b) Comparison of control and Tie2 inhibited live microvessels. ECs are shown in red and HPPs in green. HLFs are unlabeled. Scale bar is 200 µm. c) Representative histograms showing expression of Tie2 on ECs and HPPs. d) Ang1 and Ang2 expression across individual samples and e) from pooled (> *n* = 5) samples. Significance is indicated by *P < 0.05, **P < 0.01, ***P < 0.001, ****P < 0.0001, *****P < 0.00001, with one‐way ANOVA and Tukey test.

Angiopoietin and its receptor Tie2 has also been implicated in vascular regulation of the placenta,^8^ however signaling is strongly associated with the VEGF pathway. In particular, Tie2‐expressing ECs are activated by pericyte expressed Ang1 to promote quiescence,^37^ whereas in the presence of VEGFA, Ang2 acts to potently promote angiogenic remodeling in inflammation by blocking Ang1‐Tie2 interactions. Specifically, Ang1 results in Tie2 phosphorylation and downstream signaling events (P13k/Akt and ERK pathways) leading to anti‐inflammatory and prosurvival pathways, whereas Ang2 is (mostly) believed to bind Tie2 antagonistically.^38^, ^39^ First, we employed both an inhibitor and a blocking antibody against Tie2 to inhibit receptor activity in the microvessels 48 h prior to imaging. Tie2 inhibition resulted in significant changes in microvessel morphology with both microvessel diameter and branch length being significantly reduced (**Figure** **5**a). Clear differences in microvessel morphology could also be observed between the larger untreated microvessels and the thin Tie2‐inhibited microvessels, where HPP‐EC colocalization was inhibited (Figure 5b). Using flow cytometry we demonstrate Tie2 is expressed by ECs and very weakly by HPPs (Figure 5c) but not on HLF, suggesting that any Tie2 activity can largely be attributed to ECs, as reported in the literature where Tie2 is strongly expressed by HUVEC.^39^ Expression by our placental pericytes corresponds with recent findings of a reciprocal role for pericyte‐EC Tie2 signaling.^11^ We also examined whether notch inhibition with 1 × 10^−9^ m LY3039478 would affect microvessel remodeling, as it has recently been implicated in placental vascular maturation,^40^ however no effect was observed in morphological parameters (Figure 5a).

Next, we examined Ang1 and Ang2 expression in the presence or absence of functional Tie2 receptors to see if pericytes influenced the balance of these key cytokines. We also measured Ang1/2 in the presence of exogenous VEGF given the interaction between these two pathways (Figure 5d). Angiopoietin‐1 and ‐2 critically regulate EC‐pericyte crosstalk,^11^ but these cytokines were not assessed in the cytokine array. Furthermore, these cytokines are proposed to play a central role in PE pathology.^41^, ^42^ Ang1 is secreted specifically by pericytes, and to a lesser degree by other stromal cells.^43^ In contrast, Ang2 is specifically secreted by inflamed endothelial cells. Here, Tie2 inhibition significantly reduced Ang1 expression, whereas VEGFA significantly increased the expression of Ang2. In comparison to the control, Tie2 inhibition and VEGFA addition both significantly increased the ratio of Ang2/Ang1, as measured from individual samples in a single experiment (Figure 5d). These same trends were observed for pooled samples, but with increased variability between experiments (Figure 5e). Overall our results indicate that interfering with Tie2 signaling (on ECs and/or HPPs) results in loss of pericyte coverage and Ang1, which is necessary to promote vessel stability. Angiogenic factors (Ang2 and VEGFA) typically increase during placental growth and decrease toward the final stages of pregnancy; however, this phenomenon is not observed in women with PE^41^), which could implicate dysfunctional EC‐pericyte interactions.

### 2.4 Pericytes Increase PE‐Associated Cytokine Production and Microvascular Leakage

**Figure 6:**
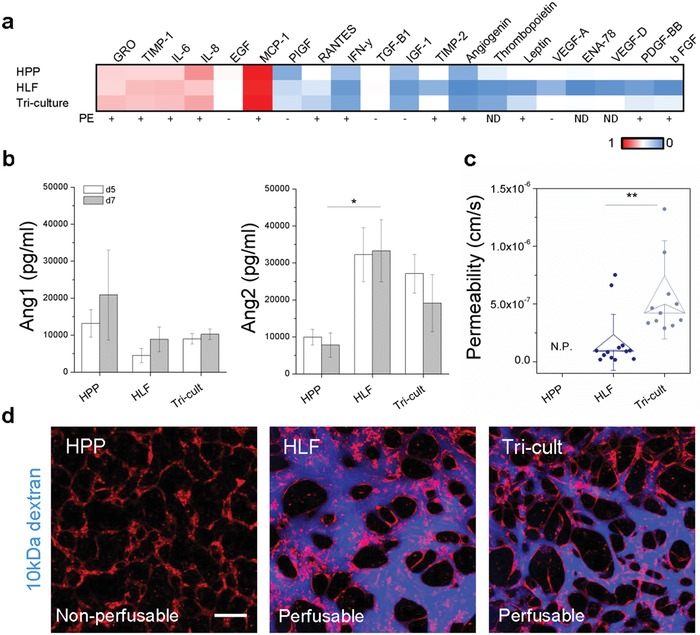
Pericytes influence PE‐affiliated cytokine expression and endothelial barrier function. a) Cytokine expression is shown for HPP, HLF, and triculture microvessel supernatants collected at day 5. HPPs result in increased PE‐associated cytokine expression, as indicated by the last row demonstrating those that are up (+) and down (−) regulated in PE. ND—no‐data in found. Here red values are high, blue low, and white are mid‐level (0.5). All cytokines were normalized to numbers between 1 and 0 based on maximum and minimum intensities from the cytokine array (Figure S4b, Supporting Information). b) Ang1/2 expression analyzed by ELISA for co‐ and tricultures, measured from pooled samples (*n* = 5). c) Permeability of microvessels perfused with 10 kDa dextran (blue) at day 7 for co‐ and tricultures. Shown is mean ± s.e.m. Significance is indicated by *P < 0.05, **P < 0.01, using *t*‐test. d) Confocal images demonstrating perfusability of HLF cocultures and Tricultures, and lack of perfusability in HPP cocultures. HUVEC—red, 10 kDa dextran–blue. Scale bar is 200 µm.

Following examination of signaling pathways important in microvascular growth, we investigated the potential secretory microenvironment generated by the presence of pericytes. To explore whether pericytes influence the cytokine milieu in the absence of GFs, supernatants were collected (pooled from *n* = 10 devices each) from co‐ and tricultures at day 5 and analyzed by cytokine array (Figure S4b, Supporting Information). Analysis showed that these microvessels expressed many cytokines often associated with inflammation in both co‐ and tricultures (**Figure** **6**a). In particular, HPPs expressed high levels of IL‐8, MCP‐1, and IFN‐y as well as VEGF, PDGF‐BB, and bFGF, many of which are upregulated in serum from women with PE (reviewed in^30^). Therefore, we further examined expression of several inflammatory cytokines by enzyme‐linked immunosorbent asasy (ELISA) (Figure S7, Supporting Information). Interestingly, TNFα and MCP‐1, but not IL‐8, were higher in HLF cocultures compared to HPP and tricultures. HLFs in coculture seemingly contribute to more proinflammatory signaling, which can be reduced by addition of interleukin‐10 (IL‐10, an anti‐inflammatory cytokine) in our system (Figure S7, Supporting Information). IL‐10 is often decreased in pre‐eclamptic patients.^44^ These results suggest that the interplay of stromal cells has a significant effect on the inflammatory response, which is in line with the observation that inflammatory signaling is often upregulated in placental‐derived stromal cells from PE patients.^45^ Nevertheless, stromal cells are not the only source of inflammation. In one study, increased IL‐6 and TNF‐α was shown to be independent of placental regulation, leading the Authors to suggest activation of leukocytes or the endothelium as potential contributors to the inflammatory PE circulatory profile.^46^

Given their role in inflammation,^38^ and our findings highlighting the significant role of Angiopoietins in EC‐pericyte signaling, we again examined Ang1/2 expression to confirm the roles of each respective stromal cell. Here, we have focused on the soluble component of Ang1 due to its prevalence in placental pathology,^42^ and did not assess the membrane‐bound protein on individual cells due to complexities in its measurement, as well it may not truly reflect the subcellular localization in our 3D culture. Pooled supernatants were collected from devices on days 5 and 7 of culture and measured by ELISA for co‐ and tricultures (Figure 6b). While HPP cocultures presented high expression of Ang1, variability was high between experiments. In contrast, HLF cocultures resulted in significantly higher Ang2 expression. Increased Ang2 and inflammatory cytokine secretion in pericyte containing tricultures (as opposed to HPP cocultures) suggested that endothelial barrier integrity may also be affected as these processes often occur concurrently.^47^

Transendothelial transport of molecules and leukocytes is typically a tightly regulated process—one that becomes dysregulated in women with PE, as demonstrated by intravascular leakiness (reviewed in 48) and increased neutrophil activation in vivo,^49^ respectively. Disrupted EC junctions have also been shown in umbilical (fetal) vessels following parturition from PE‐afflicted mothers.^50^ We exploited our system of microvessels to examine the potential contribution of HPPs to endothelial barrier dysfunction by perfusion of our co‐ and triculture vessels with 10 kDa dextran, as measured similarly to our previous work.^26^ Endothelial‐pericyte interactions are typically associated with reduced vascular permeability;^7^ however, time‐lapse measurements demonstrated an increase in endothelial permeability in the presence of placental pericytes (Figure 6c), This result contrasts previous reports of human kidney pericytes reducing HUVEC permeability.^51^ However, the same authors report that inflammatory cues stimulate mural cell detachment from ECs, and also lead to disruptions in EC junctions. While we did not specifically investigate intercellular junctions, it is possible that aberrant Ang‐VEGF signaling caused by the presence of both stromal cells results in junction discontinuities. There appears to be compensatory signaling between the stromal cells, and overall the triculture vessels appear to be leaky and inflamed (Figure 6d), similar to those seen in placental PE.

### 2.5 TNFα Enhances Neutrophil Recruitment and Microvascular Leakage

**Figure 7:**
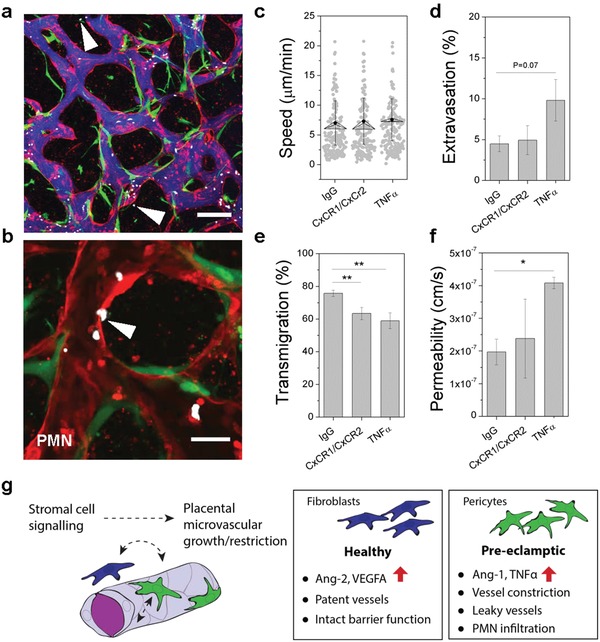
Neutrophil extravasation corresponds with microvessel permeability. a) Triculture vessels are shown perfused with neutrophils and simultaneously with 10 kDa dextran (blue). HUVEC—red, Pericytes—green, PMNs—white. Arrows indicate extravasated PMNs at ≈10 min following perfusion. Scale bar is 200 µm. b) Image of PMN (white arrow) extravasating from the lumen into the extravascular space. Scale bar is 50 µm. c–f) Measurements of PMNs exposed to IgG control antibodies, CXCR1/CXCR2 blocking antibodies, or TNFα are shown for c) mean speed, d) % extravasation, and e) % transmigration. f) Endothelial barrier function (permeability) is shown to be significantly altered by TNFα stimulation. Shown is mean ± s.e.m. Significance is indicated by **P < 0.01, ****P < 0.0001, *t*‐test. g) Cartoon demonstrating fibroblasts versus pericytes role in our triculture model.

Inflammation is associated with increased levels of TNFα and polymorphonuclear cell (PMN), or neutrophil, infiltration into the placenta in vivo (reviewed in^43^). In particular, TNFα regulates PMN chemoattractants and endothelial receptors, such as IL‐8 and ICAM‐1, which are upregulated in PE patients.^52^ Considering our triculture vessels express high levels of IL‐8, are leaky, and express high Ang2/Ang1 ratios due to the presence of pericytes, we postulated that a large number of PMNs would extravasate into the surrounding tissue in our system. First, we perfused untreated tricultures at day 7 with fluorescently labeled PMNs freshly isolated from human blood. PMNs were seen immediately within vessels following perfusion (**Figure** **7**a) and quickly extravasated across the endothelium (Figure 7b), as captured via time‐lapse imaging over short durations (≈12 min) (Video S1, Supporting Information). Microvessels were reconstructed from confocal images in 3D using Imaris, and PMNs were autotracked for speed and manually tracked for complete extravasation or transmigration (defined here as in contact with or crossing the endothelium) (Figure 7c–e).

In an attempt to reduce PMN extravasation, we included blocking antibodies against CXCR1/CXCR2 during PMN perfusion. We also utilized overnight treatment of tricultures with TNFα to see if we could enhance PMN extravasation. While there was no change in the speed of PMNs or their extravasation by blocking treatments, there was an increase for those microvessels exposed to TNFα. The increase in PMN extravasation directly corresponds with significantly increased permeability of the endothelium to 10kdA dextran in response to overnight TNFα stimulation (Figure 7f). Overall, the number of PMNs that extravasated was low, which could contribute to weak differences between antibody treatments. Notably, the % PMNs that associated with ECs was significantly reduced for microvessels treated with CXCR1/CXCR2, as would be expected (Figure 7e). Activation of PMNs by placenta‐derived growth factors (IL‐8 and syncytiotrophoblast microparticles) has been demonstrated in vitro, and increased numbers of extracellular traps are strongly associated with PE.^53^ Whether or not PMNs become activated in our preinflamed microvessels remains to be seen. Together, these results demonstrate that PMNs are recruited even in the absence of stimulation and both neutrophil recruitment and vascular leakiness is increased further by TNFα.

Our model recapitulates numerous features of placental vasculopathies, such as those seen in the placental subtype of PE, including microvessel dysfunction, inflammation, and infiltration by PMNs. However, certain limitations of the model exist. For instance, reproducing a hypoxic environment (as seen in PE) will require extensive testing to mimic the large span of physiologic oxygen tension within the placenta. Moreover, understanding the observed proliferative behavior of stromal cells is important and requires further study, since this phenomenon may contribute to vascular dysfunction. Finally, accurately assessing membrane‐bound Ang1, as opposed to the soluble form as performed herein, will provide further insight into the observed Ang1‐Tie2 reciprocal signaling between endothelial cells and pericytes. While we cannot recapitulate all aspects of the pathophysiology of placental microvasculature, nor PE, our model provides key information regarding the interplay between the endothelium and stromal cells that likely contribute to vasculopathies during gestation. From our detailed characterization of morphology and barrier function, our placental microvascular model provides strong evidence that reciprocal endothelial–pericyte signaling plays a significant role in vascular growth, the dysfunction of which could contribute to vascular degeneration in inflammatory diseases such as PE (Figure 7g). This model serves as a basis for future investigations into the role of hypoxia (which is known to drive VEGF^47^), growth factors,^54^ and hormone regulation,^43^ which all play a role in placental development and may contribute to PE. Moreover, our model will be useful for examining potential anti‐inflammatory agents (in addition to IL‐10) or other potential treatment strategies to circumvent endothelial dysfunction. Certainly, the inclusion of trophoblasts, which express a number of angiogenic factors, will also be necessary to provide a more complete picture of placental vascular development and pathology in future work.

## 3 Conclusions

A 3D model of perfusable placental microvasculature was developed using a triculture of stromal and endothelial cells. Our model is the first to generate patent vessels using placental pericytes, allowing us to examine potential mediators of vessel stabilization or dysfunction in their presence. These microvessels exhibit characteristics similar to placental pre‐eclampsia, expressing inflammatory cytokines, neutrophil recruitment, and vessel dysfunction. In this model, placental pericytes are shown to contribute to significant vessel restriction and reduced endothelial barrier function, in comparison to fibroblast cocultures. Loss of receptor function for FGF, VEGF, and Tie2 each result in significant vessel rarefaction, signifying the VEGF and Ang/Tie2 signaling pathways as crucial in regulating placental vessel growth. Tie2 binding is required to maintain vessel stability and pericyte‐association, and it mediates overall Ang1 expression—indicating reciprocal signaling between the endothelium and pericytes. Overall, we demonstrate the importance of endothelial–pericyte signaling in regulating placental vasculogenesis. In the absence of sufficient proangiogenic signaling (from fibroblasts here), placental pericytes contribute to microvessel destabilization. Dysregulation of endothelial–pericyte signaling during early placental vasculogenesis could contribute to endothelial dysfunction associated with pre‐eclampsia or other diseases such as fetal growth restriction.

## 4 Experimental Section

*Cell Culture*: HUVEC and HLF were purchased from Lonza. HUVEC were transduced to express cytoplasmic TD‐tomato (as described previously), and were used between passages 9 and 11. HUVEC were cultured in EGM‐2MV media (Lonza) on 50 µg mL^−1^ rat tail collagen I (Corning) coated T75 or T150 flasks. Unlabeled HLFs were used between passages 7 and 10 and were cultured in FGM‐2 media (Lonza) on collagen 1 coated flasks. GFP‐labeled HPP (microvascular) were acquired from Angioproteomie and were cultured in pericyte growth medium according to manufacturer's protocols. Human brain pericytes (HBP) from (ScienCell) were cultured according to manufacturer's protocols on poly‐l‐lysine coated flasks and used between passages 3 and 7. For experiments involving growth factor free media, basal media from VascuLife VEGF Medium Complete Kit (Lifeline) was used and supplemented with 1% FBS (Gibco). In one experiment, VEGF was added at the concentration used for complete media (0.5 ng mL^−1^). All cells were cultured under normal conditions at 37 °C and 5% CO_2_ in a standard incubator, dissociations were carried out using TrypLE Express (Gibco), and media was refreshed every second day.

*Device Fabrication*: Negative molds for macro devices were cut from 0.5 mm cast Clarex (polymethylmethacrylate) (Astra Products) on an Epilogue laser cutter. A rasterized pattern was made alongside the middle channel ≈¼ of the height in order to guide fibrin gel insertion (as opposed to typical posts). Thin cut molds were acetone bonded to a larger ¼” thick acrylic sheet surrounded by the same height lip in order to facilitate reproducible PDMS device fabrication. PDMS (Ellsworth Adhesives) was mixed at a 10:1 elastomer to cross‐linker ratio according to manufacturer's protocols, degassed, and poured onto mold. Following further degassing, the mold was placed in a 60 °C oven overnight. Devices were cut from the mold, and punched using 1 and 2 mm diameter biopsy punches for the gel and media ports, respectively. Devices were autoclaved in a dry cycle for 20 min prior to air‐plasma bonding (Harrick) to clean #1 thickness glass slides. Devices were coated with 1 mg mL^−1^ poly‐d‐lysine (Sigma‐Aldrich) for several hours before rinsing with sterile cell culture grade water (Lonza) and finally incubating at 80 °C for drying and returning PDMS to its native hydrophobic state.

*Device Seeding and Culture of Microvessels*: First, fibrinogen from bovine plasma (Sigma) was reconstituted in Dulbecco's phosphate‐buffered saline (DPBS) (Lonza) for several hours at 37 °C at a concentration of 6 mg mL^−1^. Fibrinogen was sterile filtered using a 5 mL syringe and 200 µm filter prior to use. Thrombin (Sigma) stock solution was made at 100 U mL^−1^ in 0.1% w/v bovine serum albumin (BSA) solution and stored at −20 °C. Thrombin was diluted in cold EGM‐2MV prior to use when seeding devices to a concentration of 4 U mL^−1^. Endothelial cells and stromal cells were dissociated and mixed in the appropriate volume of 4 U mL^−1^ thrombin solution to make up 24 m cells mL^−1^ for ECs, and 4.8 m cells mL^−1^ for stromal cells (either fibroblasts, pericytes, or a combination of the two) at a 5:1 EC to stromal cell ratio. Following mixing of EC and stromal cell solutions, aliquots for seeding 2 devices simultaneously were prepared (≈40 µL) on ice. An equal volume of fibrinogen solution to cell‐thrombin solution was added, mixed, and carefully pipetted into 1 gel inlet, allowing about ½ of the gel region to fill, prior to completely filling the gel region by the opposite inlet. Fibrinogen + thrombin mixture was allowed to polymerize in a fibrin gel in a ≈37 °C humidity chamber for 15–20 min. EGM‐2MV was added to the 2 media channels (150 uL total) and completely changed daily. For monoculture experiments, HUVECs were seeded at the same density without stromal cells, and in some cases a monolayer of pericytes (1.5 m mL^−1^) was added to one media channel at day 3 (Figure S2c–f, Supporting Information). All cultures were maintained under static conditions.

*Growth Factors and Inhibitors*: Recombinant human bFGF (Peprotech) was reconstituted in sterile water containing 0.1% BSA. Recombinant human VEGF165 (VEGFA) was reconstituted in DPBS containing 0.1% BSA. Growth factors were used at concentrations of 50 ng mL^−1^, for VEGFA and bFGF. The VEGFR inhibitor SU5416 (Sigma) was used at a concentration of 5 × 10^−6^ m. An inhibitor of PDGFR (α and β) AG 1296 (Calbiochem, EMD Millipore) was reconstituted in dimethyl sulphoxide (DMSO) and used at a concentration of 2 × 10^−6^ m. A notch inhibitor, LY3039478 (Cayman Chemical), was reconstituted in DMSO and used at concentrations of and 1 × 10^−9^ m. An inhibitor of Tie2 kinase (Cayman Chemical) was reconstituted in DMSO and used at a concentration of 5 × 10^−6^ m. A selective inhibitor of FGF‐1 receptor tyrosine kinase (PD‐166 866) (Sigma‐Aldrich) was reconstituted in DMSO and used at a concentration of 100 × 10^−9^ m. A polyclonal human Tie2 antibody was purchased from R&D (goat IgG). Devices were cultured with Tie2 ab at 50 µg mL^−1^ in serum‐free basal media (Vasculife, Lifeline) for 2 h, prior to complete media change back to basal media + 1% FBS. Exogenous IL‐10 (Peprotech) was reconstituted at 10 µg mL^−1^ in PBS (+1% BSA) and added to devices at day 3 using 150 ng mL^−1^ concentrations (daily). Recombinant Human VEGFR1/Flt‐1 Fc Chimera Protein (R&D) was used at 1 µg mL^−1^ concentrations from day 3 onward.

*Imaging and Morphology Quantification*: All images were acquired on an Olympus IX81 confocal microscope using Fluoview v4.1 software. Confocal z‐stack images were acquired for all time‐points and consisted of a 5 µm step size and ≈20–25 slices (beyond which intensity diminishes). For analysis, macros were generated to perform the following steps using ImageJ: images were converted to .tiff files, projected (maximum intensity) in the z‐direction, outliers were removed (radius of 2 pixels), smoothed using a Gaussian filter (sigma 2 pixels) and converted to binary images. The *analyze particles* and *2D skeletonize* built‐in ImageJ functions were used to measure area and branch geometry using custom automated macros, respectively.

*Cytokine Analysis*: Supernatants were collected from *n* = 10 coculture (HPP and HLF) devices at days 3 and 5 and were frozen until use. A human angiogenesis antibody array (Abcam) was used according to manufacturer's protocols. The same array was used to compare day 5 analytes collected from *n* = 5 tri‐ and coculture devices, from 2 independent experiments. Separate Ang‐1 (ThermoFisher) and Ang‐2 (Invitrogen) ELISA kits were used as directed to measure cytokine production from either pooled supernatants (collected from three devices per biological repeat) or from individual samples, as indicated. MCP‐1 and IL‐8 instant ELISA kits (ThermoFisher), as well as a TNFα ultrasensitive ELISA kit (Invitrogen) was used to measure cytokine expression from individual samples.

*Permeability Measurements*: Briefly, cells were grown in co‐ or triculture in the microfluidic devices until day 4, following which a monolayer of ECs was seeded into both media channels at a concentration of 1.5 m cells mL^−1^. At day 7, devices were perfused with 10 kDa anionic Cascade‐blue dextran (ThermoFisher) by a temporary drop in pressure across the gel region (complete removal of media followed by introduction of 40 µL of fluorescent solute into one channel). Once the microvessels are fully perfused, flow is stopped by applying an equal volume of dextran solution (40 µL) in the opposing channel. See the recent paper demonstrating this process in detail.^27^ Following stabilization (≈2–3 min), time‐lapse confocal volumes were captured (3 × 5 min intervals). Maximum projection images (Dextran channel at *t* = 0) were used to generate a binary outline of the vessel perimeter (*p*_v_) and extravascular tissue area (*A*_T_) in ImageJ. Assuming that intensity (*I*) is linearly related to the fluorophore concentration, flux across the imaging boundary is negligible, and transendothelial flux is constant, the permeability *P*(cm s^−1^) of the microvessels to solutes can be approximated, as in Equation (1) as it is done previously:^26^

(1) $$P\;\left(t\right)\;=\;\frac{A_{T}\left(I_{T_{f}}-I_{T_{0}}\right)}{p_{v}\;t\;\left(I_{V_{0}}-I_{T_{0}}\right)}$$

*Neutrophil Isolation and Extravasation*: Human neutrophils were freshly isolated from human blood collected from healthy donors (informed patient consent and samples collected from Research Blood Components, Cambridge, MA). Blood was anticoagulated with sodium citrate and mixed 1:1 with Hank's balanced salt solution (HBSS) (Invitrogen). Blood with HBSS was layered onto Histopaque 1077 (Sigma), and centrifuged for 30 min at 1400 RPM. Plasma and peripheral blood mononuclear cell layers were removed by further separating neutrophils using a density gradient with 2% dextran from Leuconostoc spp. (Sigma) for 30 min at 1400 RPM, followed by red blood cell lysis with cold sterile water. Isolated cells were stained with Cell Tracker Deep Red (Invitrogen) at 0.5 × 10^−6^ m for 10 min, washed twice with HBSS, and resuspended at 6 m mL^−1^ in HBSS. All isolation procedures were done at room temperature.

PMNs were subsequently treated with CXCR1 and CXCR2 antibodies (10 ug mL^−1^ each), the control IgG antibody at the same concentration, or no treatment, and left on ice for 30 min. Following incubation, neutrophils (6 m mL^−1^) were mixed 1:1 with 10 kDa blue dextran in complete media and perfused into networks (as done for permeability measurements). In some cases, microvessels were treated with 10 ng mL^−1^ TNFα (Peprotech) overnight prior to neutrophil perfusion. Time‐lapse measurements were taken (5 × 2 min intervals) to capture neutrophil migration speeds and corresponding EC permeability. Samples were fixed with 4% paraformaldehyde (PFA) ≈20 min postimaging. Imaris v.8.4.1 (Bitplane) was used to track PMNs semiautomatically from time‐lapse images. Fixed sample images were used (10x ROIs) to generate surfaces in Imaris from fluorescent ECs and PMNs, in order to manually locate those PMNs that appeared in the extra‐vascular space (extravasated) and those touching the surface (transmigrating).

*Immunofluorescence Staining*: Cells were fixed with 4% PFA for 20 min prior to washing with DPBS. Samples were solubilized using 0.1%TX, rinsed with PBS, and incubated in appropriate blocking buffer (containing BSA and serum from source of secondary antibody) for >2h. Primary antibodies were diluted in wash buffer (0.5% BSA in DPBS) and added to samples overnight at 4C. The following day, samples were washed with wash buffer and then incubated with the appropriate secondary antibody and counter stains for ≈4 h. Samples were washed with PBS and imaged immediately. To stain within the devices, a pressure gradient was applied across the gel and generally incubation times were increased. Antibodies against NG2 (rabbit), PDGFRβ (mouse), αSMA (rabbit), and laminin (rabbit) were purchased from Abcam.

*Flow Cytometry*: Co‐ and tricultures were seeded and maintained for 5 d prior to resecting the gel from the device. The gel was digested with a combination of Accutase (Innovative Cell Technologies) and 50 FU mL^−1^ Nattokinase (Japan Bioscience Ltd) prior to filtration through a 100 µm pore filter, and then analyzed by flow cytometry (BD LSR II HTS‐2). For analysis of Tie2 expression, cells (HUVECs, HPPs, and HLFs) were cultured in T75 flasks, dissociated, and stained on ice for 20 min with human Tie2 antibody (R&D), followed by secondary antibody staining. FlowJo v.10 software was used for gating and analysis. The same methods as outlined above were used for 2D and 3D investigation of smooth muscle and pericyte‐specific markers anti‐αSMA (Abcam, ab32575), and anti‐NG2 (Abcam, ab129051), respectively. Biolegend intracellular staining buffer (Cat# 421 002) was used for preparation of intracellular staining of anti‐Calponin (Novus Sheep pAb, Cat AF7900‐SP), and Biolegend anti‐PDGF‐RB ab (clone 18A2).

*Statistics*: Results shown are mean ± s.e.m., except for box plots which demonstrate s.e.m at the box top and bottom and SD as bars. Comparison between all drugged groups was performed using one‐way ANOVA, with *p* < 0.05 treated as significant, indicated by subsequent Tukey means comparison tests using OriginPro 8. Where appropriate, student's *t*‐tests were performed. All data shown are for at least 2 separate experiments with *n* ≥ 3 devices each, with 3 measurements made for each device, except in the case of permeability (2 measurements).

## Conflict of Interest

R.D.K. has financial interests in Aim Biotech.

## Supporting information

Supplementary

Supplementary
