# Supplementary material for: Pericytes Contribute to Dysfunction in a Human 3D Model of Placental Microvasculature through VEGF‐Ang‐Tie2 Signaling
Source: Adv Sci (Weinh). 2019 Oct 29;6(23):1900878. doi: 10.1002/advs.201900878 (PMC6891921; doi:10.1002/advs.201900878)
Supplement: Supplementary file 1 — Supplementary [file ADVS-6-1900878-s002.pdf]

Copyright WILEY-VCH Verlag GmbH & Co. KGaA, 69469 Weinheim, Germany, 2019.

ADVANCED SCIENCE Open Access

Supporting Information

for *Adv. Sci.,* DOI: 10.1002/advs.201900878

Pericytes Contribute to Dysfunction in a Human 3D Model of Placental Microvasculature through VEGF-Ang-Tie2 Signaling

Kristina Haase, Mark R. Gillrie, Cynthia Hajal, and Roger D. Kamm**

Supporting Information

Pericytes Contribute to Microvascular Dysfunction in a Human 3D Model of Placental Microvasculature through VEGF-Ang-Tie2 Signaling

*Kristina Haase, Mark R. Gillrie, Cynthia Hajal, and Roger D. Kamm**

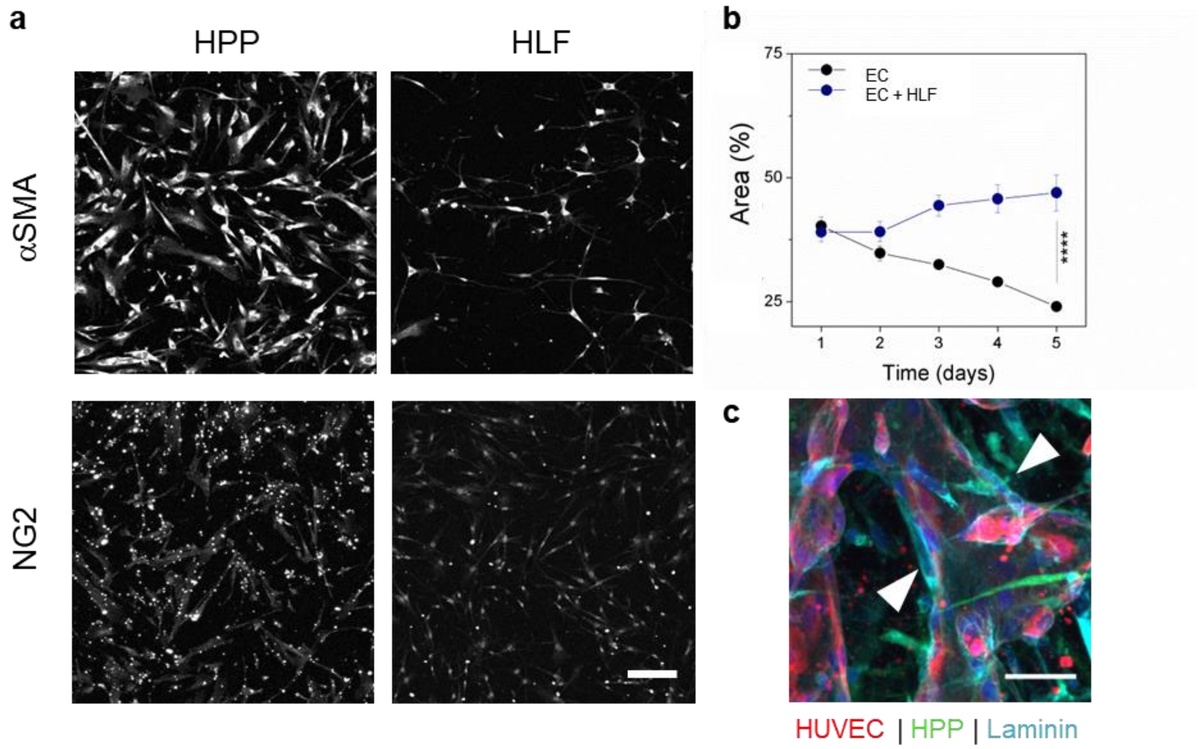

Figure S1. Stromal cell interactions affect vascular growth**.** a) IHC of placental pericytes (HPP) and human lung fibroblasts (HLF) demonstrating their expression of common stromal cell markers. Scale bar is 200 microns. b) A comparison of vascular growth (measured by vessel area coverage) between HUVEC mono-cultures and fibroblast co-cultures. Shown is mean ± s.e.m. for n=4 devices each. ****P < 0.0001 with t-test at day 5. c) Fixed image of placental pericytes embedded in the basement membrane (indicated by white arrows) of endothelial vessels. HUVEC (red), laminin (cyan – false colored), and pericytes (green). Scale bar is 50 microns.

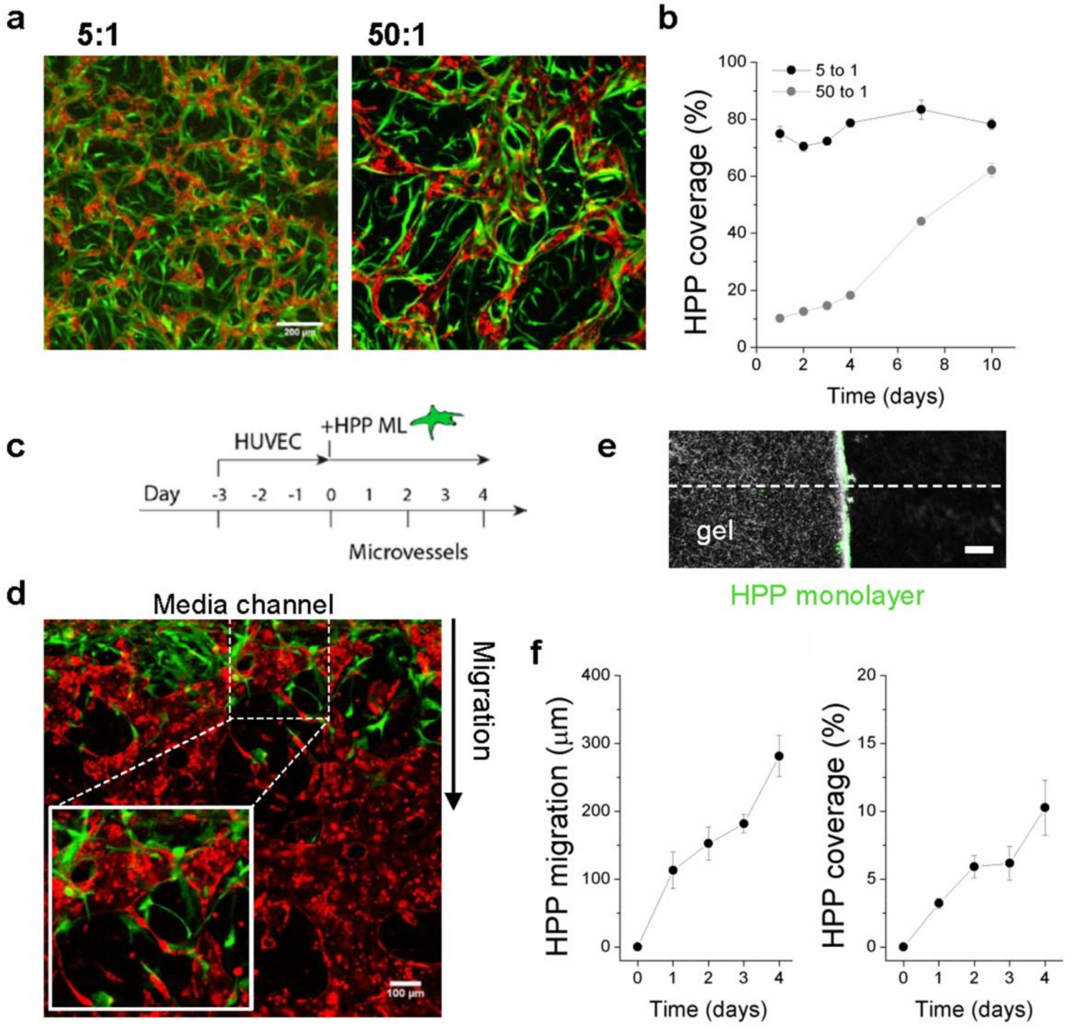

Figure S2. Placental pericytes proliferate and are recruited by ECs**.** a) HPPs (green) seeded at different ratios in HUVEC (red) co-cultures. Most pericytes wrap around or appear adjacent to vessels. Scale bar is 200 microns. b) Area coverage of HPPs increases over 10 days in the co-culture, independently of the initial seeding density. c) Schematic of the co-culture, where HUVEC are cultured alone in 3D, followed by addition of an HPP monolayer to one side of the hydrogel. d) Confocal image at day 4 demonstrating invasion of HPPs into HUVEC microvessels. Scale bar is 100 microns, with inset 1.5x. e) Reflectance image of fibrin hydrogel and HPP monolayer after 4 days in culture. Minimal HPP invasion occurred without HUVEC. f) Migration and % coverage from live confocal images, as shown in (d), were tracked for HPPs over several days. Shown is mean ± s.e.m for n=3 devices.

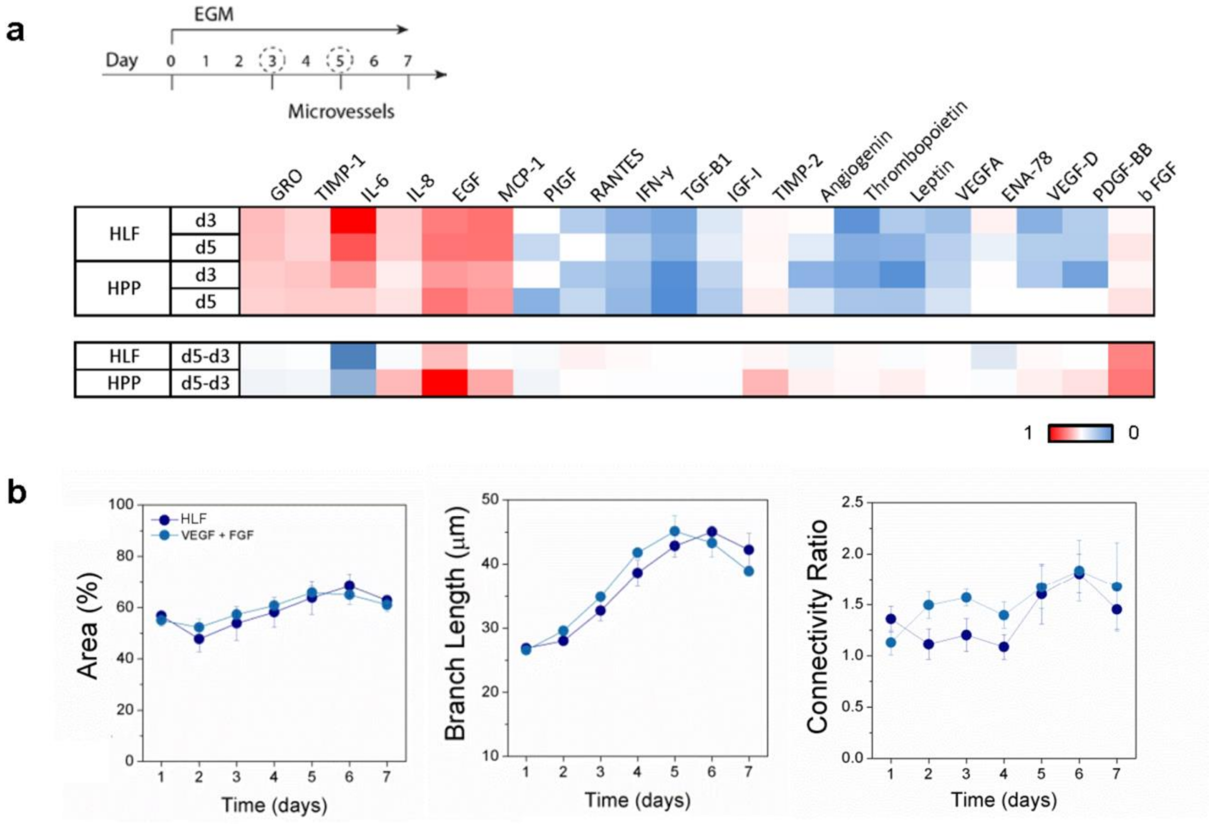

Figure S3. Stromal-cells influence cytokine expression over time. a) Schematic of collection times and cytokine array heat map for HLF and HPP co-cultures. Supernatant was collected from n=10 devices each (pooled). Representative colors (normalized) are from 2 repeats on the same membrane. Shown below is the difference between day 5 and 3 for each co-culture. b) Vessel growth is shown for untreated and GF-treated HUVEC-HLF co-cultures. No change in the parameters occurred due to the presence of VEGFA and bFGF. Shown are mean ± s.e.m. for ≥ n=3 devices each.

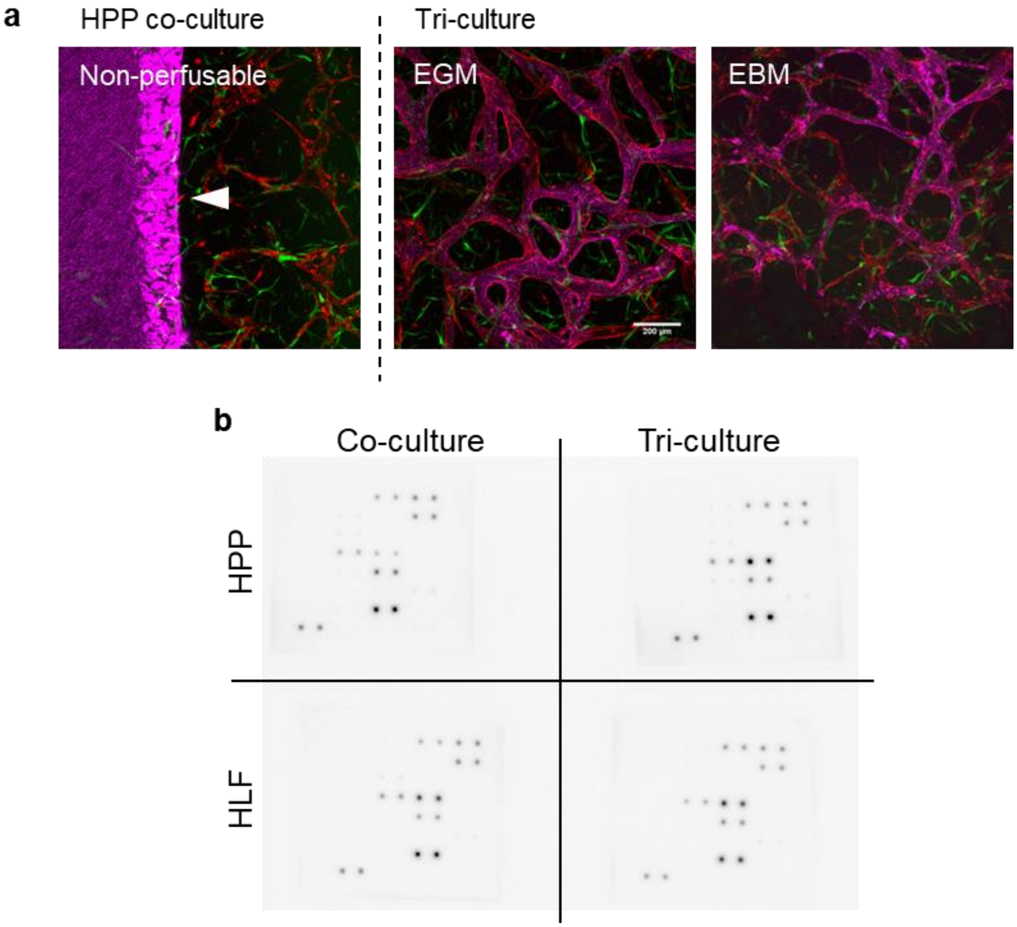

Figure S4. Tri-culture vessels are largely influenced by growth factors. a) Representative (live) confocal images demonstrating non-perfusable HPP vessels and perfusable tri-culture vessels grown in full GF media (EGM) and basal media (EBM). HUVEC (red), HPPs (green), 10 micron beads (magenta). White arrow represents bead accumulation at media channel and gel interface. b) Cytokine array membranes exposed using chemo-luminescence detector (ImageQuant LAS 4000 for ~12 mins).

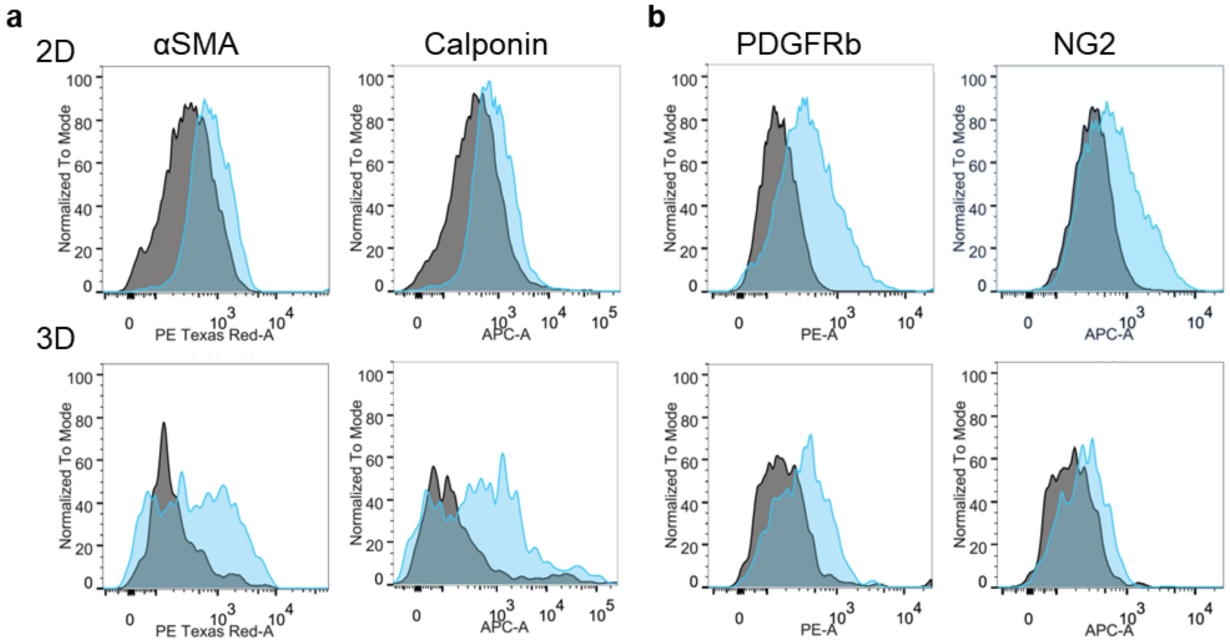

Figure S5. Characterization of pericytes from monoculture (2D) versus 3D co-cultures. A) Pericytes express smooth muscle markers αSMA and Calponin in 2D, and appear to be increased following 3D culture. B) Expression of pericyte-specific markers PDGFRb and NG2 is also shown in 2D and 3D. Expression levels are weak in 3D due to difficulties in gel digestion (containing also HUVECs and HLFs) and preparation of over-expressing GFP-labelled pericytes. Samples were dissected and pooled from a T25 flask and 3 devices for 2D and 3D analysis, respectively.

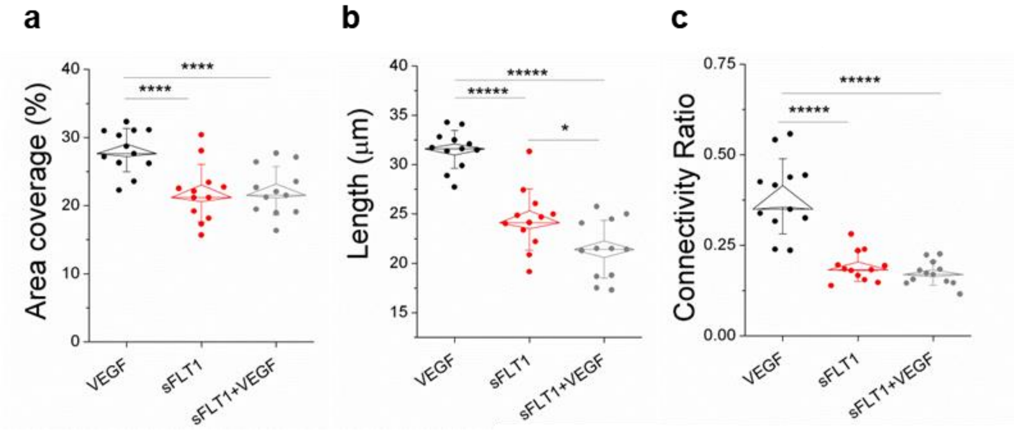

Figure S6. sFLT1, a soluble receptor for VEGF, significantly contributes to impaired vessel growth. Tri-culture vessels are compared on day 7 of culture after 4 days of treatment with 50ng/mL VEGF, 1ug/mL of sFLT-1, or a combination of the 2 soluble factors in basal media + 1%FBS. Morphology was significantly altered in the presence of sFLT1, as seen by changes in a) vessel area coverage, b) mean vessel length, and c) connectivity ratio (vessel junctions/endpoints). N=3 samples each with 3 measured regions for each sample. Significance is shown using a t-test between samples with * P<0.05, and **** P<0.0001, *****P<0.000001.

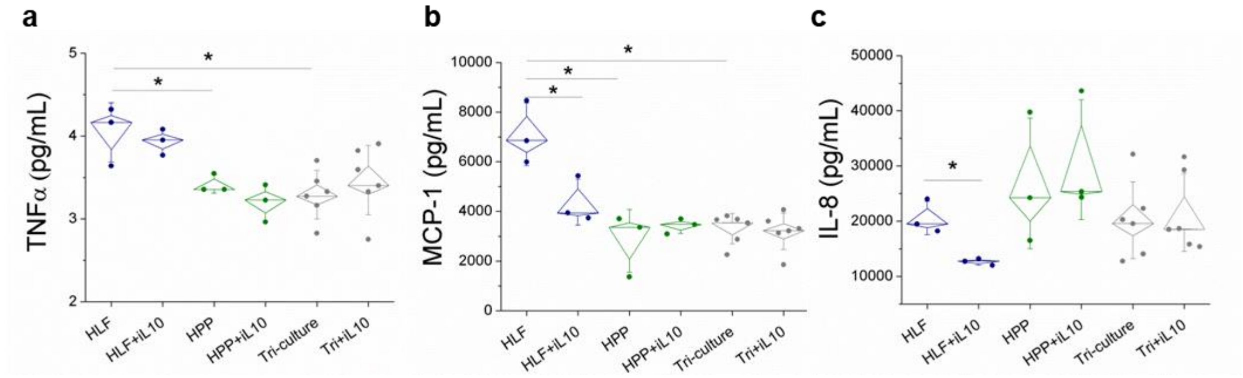

Figure S7. Inflammatory cytokine expression measured from co- and tri-culture supernatants. Expression levels were measured by ELISA for HLF and HPP co-cultures, as well as the tri-culture vessels, at day 5 for a) TNFα, b) MCP-1, and c) IL-8. Significance is indicated by * P<0.05, using t-test. N ≥ 3 samples each.
